# Direct Massage of the Heart in Chloroform Poisoning

**Published:** 1901-02-09

**Authors:** 


### DIRECT MASSAGE OF THE HEART IN CHLOROFORM POISONING.

A case of death from chloroform occurred some time ago in the practice of Dr. Maag, in which direct massage of the heart, combined with artificial respiration, was tried. The case, which is one of very great interest, was reported in the Danish journal *Hospitalstidende*, and an abridged translation has recently been published by Dr. Freyberger.[1] The method referred to was invented by Dr. Prus, of Lemberg. A labourer, aged 27 years, was admitted to hospital suffering from sciatica, which had troubled him on and off for nine months. Medicinal treatment having proved ineffective, Dr. Maag proposed to stretch the sciatic nerve. Chloroform was administered by a hospital attendant, under the direction of the doctor, an ordinary drop bottle and an Esmarch's mask being used. On the incision being made the patient struggled violently, he was given more chloroform when he suddenly became asphyxiated. From this under appropriate treatment he recovered and the operation was proceeded with without further anæsthetic, but after a few minutes the breathing again stopped. After about ten minutes spent in applying artificial respiration, etc., the trachea was opened, a soft indiarubber tube was passed into the trachea and air blown through it, but the patient remained asphyxiated and pulseless. Thereupon Dr. Maag resolved as a last chance to try direct massage of the heart after Dr. Prus' method. "An oblong skin-muscle flap was formed which had its base in the left mammillary line, and whose borders ran parallel to the third and fifth ribs and the left sternal edge respectively. The third and fourth ribs were then detached as close to the sternum as possible; pieces of the ribs, measuring 2½ inches in length, were resected and reflected with the skin-muscle flap. It was then seen that the left pleural cavity had inadvertently been opened. The right hand was introduced through the opening and the heart grasped without opening the pericardial sac. No contraction could be felt." After a few rhythmic compressions, however, the heart began to contract independently; and it would appear from the account that by dint of repeating the compression whenever the heart failed and repeating the inflations whenever the respiration flagged the patient was kept "alive" for eleven hours and a half! He never recovered consciousness however. Commenting on this case Dr. Freyberger says that the interest of the case centres in the extraordinary, if not perfectly unique, effect produced by direct massage of the heart. "A person under chloroform becomes asphyxiated. All known methods of reanimation fail; the body lies on the operating table without pulse and respiration, cold and motionless. All visible manifestations of life are gone. A couple of ribs are resected, and the heart is exposed to view; it is a flaccid, lifeless, muscular pouch. The heart is then grasped and rhythmically compressed while air is being blown down the trachea. After a little while the latent vitality of the cells of the heart muscle is again stimulated into action; the heart contracts. Feeble at first and tentative, as it were, appears the recurrent action of the heart; but the organ soon regains strength, and makes the blood circulate. Some time later the first gasping respiration takes place, then a second and a third, and life has practically been restored." Such is the story. Had the left pleura not been inadvertently opened it seems just possible that the patient might have been saved. But with one lung partially adherent, as was shown post-mortem, and with a pneumo-thorax on the other side his chances were small.

[1] Treatment, January, 1901.

